# Early detection and management of hearing loss to reduce dementia risk in older adults with mild cognitive impairment: findings from the treating auditory impairment and cognition trial (TACT)

**DOI:** 10.1093/ageing/afaf004

**Published:** 2025-01-21

**Authors:** Ruan-Ching Yu, Menelaos Pavlou, Anne G M Schilder, Doris-Eva Bamiou, Glyn Lewis, Frank Robert Lin, Gill Livingston, Danielle Proctor, Rumana Omar, Sergi G Costafreda

**Affiliations:** Division of Psychiatry, University College London, London, UK; Department of Statistical Science, University College London, London WC1E 6BT, UK; Ear Institute, University College London, London, UK; National Institute for Health Research (NIHR) University College London Hospitals (UCLH) Biomedical Research Centre, UCLH National Health Service (NHS) Foundation Trust, London, England, UK; Royal National Ear, Nose, Throat (ENT) and Eastman Dental Hospitals, UCLH NHS Foundation Trust, London, UK; Ear Institute, University College London, London, UK; Division of Psychiatry, University College London, London, UK; North London NHS Foundation Trust, London, UK; Department of Otolaryngology, The Johns Hopkins University School of Medicine, Baltimore, MD, USA; Division of Psychiatry, University College London, London, UK; North London NHS Foundation Trust, London, UK; North London NHS Foundation Trust, London, UK; Department of Clinical, Educational and Health Psychology, University College London, London, UK; Department of Statistical Science, University College London, London WC1E 6BT, UK; Division of Psychiatry, University College London, London, UK; North London NHS Foundation Trust, London, UK

**Keywords:** randomised controlled trial, mild cognitive impairment, hearing loss, cognition, dementia, older people

## Abstract

**Background:**

Age-related hearing loss and mild cognitive impairment (MCI) independently increase dementia risk. The Ageing and Cognitive Health Evaluation in Elders randomised controlled trial (RCT) found hearing aids reduce cognitive decline in high-risk older adults with poor hearing.

**Methods:**

This pilot RCT in London memory clinics randomised people with MCI (aged ≥55, untreated hearing loss defined as Pure Tone Average 0.5–4 KHz between 25–70 dB) into two groups. The intervention group received 4 sessions of hearing aid fitting and support. The control group received healthy ageing education and a GP letter recommending audiological referral. Both were followed for 6 months. Primary outcomes were recruitment (feasibility target: 50%; 95% CI: 39%–61%) and retention (feasibility target: 80%; 95% CI: 71%–89%); intervention completion (≥2 visits) and hearing aid use (acceptability target: 80%; 95% CI: 71%–89%) for the intervention group and 50% difference between arms (95% CI: 31%–69%). Secondary outcomes included hearing aid fitting, cognition and other measures.

**Results:**

From October 2018 to March 2020, 58 participants were recruited (29 per group, 95% [86%–99%]). Twenty-four participants were fitted with hearing aids in the intervention arm, and 6 in the control arm (difference: 62% [42%–82%]). At 6 months, retention was 81% [69%–90%]. Hearing intervention completion (≥2 visits) was achieved by 24 (83%). Daily hearing aid use was reported by 18 (75%) intervention versus 5 (22%) control participants, a difference of 53% [29%–77%].

**Conclusion:**

Randomisation of people with MCI to a personalised hearing intervention versus control is feasible. These findings support proceeding to a fully-powered multicentre RCT.

## Key Points

- Feasibility trial of implementing a personalised hearing intervention in people with mild cognitive impairment (MCI) and hearing loss was successful.
- The intervention has potential to improve hearing aid use in people with MCI and hearing loss.
- This study provides insights for future randomised studies of hearing interventions to mitigate cognitive decline.

## Introduction

Dementia and hearing loss are global health concerns, affecting more than 55 million [1, 2] and 1.5 billion [3] people worldwide, respectively. The Lancet Commission on Dementia Prevention, Intervention and Care summarised the most recent research and suggested that treating hearing loss could be an effective strategy to preserve cognitive function and potentially reduce the risk of dementia [4, 5]. A recent meta-analysis estimates that hearing aid use may reduce the rate of cognitive decline by 19% [6]. The Ageing and Cognitive Health Evaluation in Elders (ACHIEVE) study, the first large-scale randomised controlled trial (RCT) of a 3-year hearing aid intervention in cognitively healthy adults aged 70 to 84, found no effect on cognitive function across the total study cohort which comprised two distinct study populations [7, 8]. However, in a pre-specified analysis of the study population at higher baseline dementia risk, including lower baseline cognition and older age, hearing intervention reduced cognitive decline by 48% [7, 8]. This calls for further research in people at high risk of dementia.

Mild cognitive impairment (MCI) is a condition that exists between normal age-related cognitive changes and dementia, affecting up to one in five older adults [9]. Adult-onset hearing loss, which impacts 10%–31% of people over the age of 55 [10], increases the risk of developing MCI by 44% [11]. Both hearing loss and MCI are independently associated with an increased risk of progressing to dementia [5]. Beyond cognition, untreated hearing loss has been linked to lower overall quality of life, poorer psychological wellbeing and depression, and hearing aids may mitigate these detrimental effects [12].

However, hearing aid uptake and consistent use is low, with only 11% of people worldwide who would benefit from hearing aids getting them [13]. In the UK, where the NHS provides free hearing aids, hearing aid adoption rates amongst those aged 55 and over with self-reported hearing loss ranged from 43% to 65% in 2022 [10]. These figures are based on self-report in an online panel survey and likely include few people with MCI.

We developed a home-based personalised hearing intervention specifically designed for people with MCI. The intervention involved four sessions, including hearing aid fitting and tailored support to encourage consistent use and optimise communication strategies [14–16]. We piloted it against a control group receiving education on healthy ageing as well as a recommendation letter with the results of their audiological assessment for their GP to consider a referral to audiological services. The primary aim of our pilot study was to establish the feasibility of carrying out a fully-powered RCT. We also collected pilot data to inform this fully-powered RCT.

## Methods

### Recruitment and eligibility

The study was approved by London—Surrey Research Ethics Committee (REC reference 18/LO/1196, IRAS ID 246188) in August 2018, and retrospectively registered with ISRCTN in February 2020 (IRAS ID 246188, https://www.isrctn.com/ISRCTN47591507). Participants were recruited from National Health Service (NHS) Community Memory Services across three participating NHS London trusts.

**Figure 1:**
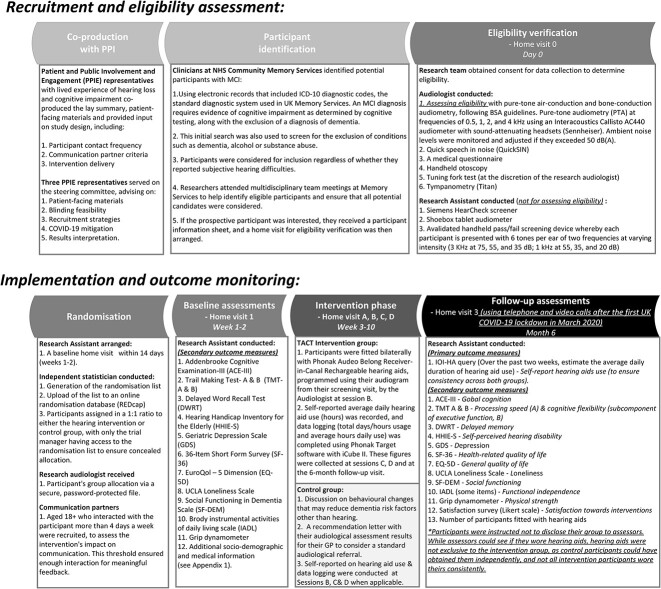
Flowchart of study assessments and procedures.

The treating auditory impairment and cognition pilot trial (TACT) was a 1-year, multicentre, parallel-group, feasibility study of an RCT. We included people aged 55 years and over with an ICD-10 diagnosis of MCI [17] as recorded by their Memory Service, residing in the community (not hospitalised or living in care homes), having adult-onset hearing loss (defined as a better-ear pure tone average [PTA] of 0.5–4 kHz thresholds between 25–70 decibel Hearing Level [dB HL], or better-ear 4 kHz threshold ≥30 dB HL) [18], with a Phoneme Recognition in Quiet (PRQ) score of ≥60% in the better hearing ear [19, 20],, and having mental capacity to provide informed consent to trial procedures, as evaluated by Good Clinical Practice (GCP) trained investigators. We excluded participants with a history of childhood onset hearing loss, evidence of conductive hearing loss as determined by the study audiologist based on PTA, tympanometry and tuning fork test (at the discretion of the research audiologist) and those who, in the opinion of the audiologist, met criteria for onward medical referral [21], or who had used hearing aids within the past month. Additionally, those with occluding cerumen were temporarily excluded until they could arrange for its removal, and participants with a current diagnosis of alcohol or substance use disorder based on ICD-10 criteria were excluded [17]. If available, we also offered participation to communication partners. Face-to-face recruitment for the trial had to stop prematurely in March 2020 due to COVID lockdown when 58 participants (out of total target of 76) had been recruited and randomised. Intervention support and follow-up continued remotely in accordance with guidance from Medicines and Healthcare products Regulatory Agency (MHRA) [22]. In July 2020, we obtained ethical approval for an additional remote intervention for a non-randomised additional sub-study, where we recruited participants that could not be randomised into the TACT trial due to COVID lockdown, and provided hearing aids and remote support for use. These study participants are separate from the ones whose results are presented in this paper. Further details on the procedures for participant identification, recruitment and the criteria of communication partners are provided in Fig. 1 (flowchart).

### Procedures

Eligibility was assessed by a research audiologist using pure-tone air-conduction and bone conduction audiometry, following BSA guidelines [23]. Ambient noise levels were monitored, and adjustments were made if levels exceeded 50 dB(A).

For participants who met the inclusion and exclusion criteria, a baseline home visit by a research assistant (RA) was arranged within 14 days (weeks 1–2). During this visit, additional socio-demographic and medical information were collected, and neurocognitive tests and social functioning scales were administered (see Fig. 1 and Appendix 1 in the Supplementary Data section for the full details of the assessments used in the study)*.* Once eligibility was confirmed, the research audiologist received the participant’s group allocation via a secure, password-protected file.

In addition, all participants, including those not eligible for randomisation, had hearing screening with Hearcheck and Shoebox audiometers, from an RA. These were exploratory and not used for eligibility in this trial but to assess their potential use in future trials. We will report the diagnostic validity and acceptability of these screens in a separate publication.

### Interventions

Details on the intervention and its co-production with patient and public involvement and engagement are given in Fig. 1.

#### Hearing intervention—treating auditory impairment and cognition pilot trial

The TACT hearing intervention was based on the intervention developed for the ACHIEVE trial [7, 8], adapted for the UK setting according to British Society of Audiology practice guidance and National Institute for Health and Care Excellence (NICE) guidance for hearing assessment and hearing aids fitting [24, 25]. See Appendix 2 and 3 in the Supplementary Data section for the full details of the intervention. In brief, four sessions included: A. orientation and goal setting, using the Client Oriented Scale of Improvement questionnaire [26] to focus on individual communication goals, including priorities and challenges, B. hearing aid fitting and training, with aids tailored to participants’ audiological profiles and integrated into daily routines, C. review of hearing aid use and communication strategies, where experiences were assessed, and adjustments made based on their feedback, and D. hearing aid optimisation and progress review, refining hearing aids settings and communication strategies based on ongoing experiences. Sessions B and D were conducted by a research audiologist and Sessions A and C by an RA trained in study procedures. Participants were encouraged to contact the trial team for additional support, ensuring continuous personalised support throughout the trial. Our intervention to support hearing aid use is significantly more comprehensive than what is typically provided in standard audiology practice.

Following the first UK COVID-19 lockdown in March 2020, delivery of the home-visit interventions was stopped, with support provide remotely following Ethics approval.

#### Control group

The control group received a healthy ageing education based on the successful ageing intervention used in ACHIEVE [7, 8] with modifications being made for the UK context and to follow NHS guidance [25], to match the intensity of study contact of the TACT hearing intervention group (Appendix 3 in the Supplementary Data section for the full details of the intervention). In brief, participants in the control group received four scheduled home visits with an RA, discussing behavioural changes that may reduce dementia risk factors other than hearing. These included: hypertension, diet, physical activity and healthy bones, joints and muscles. Following standard referral pathways and with the participant’s consent, the research team wrote to the participant’s GP with the results of their audiological assessment and recommendations to consider a referral to audiology services, if appropriate.

### Outcomes

The primary goal of the trial was to establish readiness for a future fully-powered RCT, based on whether the pilot trial procedures were effective in ensuring recruitment and retention, and whether interventions were feasible and acceptable. Feasibility was defined as proportion of eligible participants being recruited and retained in the trial for 6 months, acceptability of the hearing intervention was assessed by the proportion of participants in the hearing intervention group who successfully completed the intervention, and by the difference in self-reported daily use of hearing aids between the groups at the 6-month follow-up visit. Secondary outcomes included various measures listed in Fig. 1.

RAs blinded to participants’ group allocation collected outcomes at 6 months post-randomisation and were not involved in intervention delivering.

Details on sample size calculation and the goals set in the study are provided in Appendix 4 in the Supplementary Data section.

### Statistical analyses

Statistical analysis was performed using Stata version 16, by a statistician not blinded to participant’s allocation. Counts and proportions for categorical variables and mean/medians with standard deviation (SD)/Interquartile Range (IQR) for continuous variables, were used to summarise the baseline characteristics of the intervention and control groups. The outcomes of recruitment rate, randomisation acceptability, retention level and adherence were estimated using proportions and 95% confidence intervals. P-values were not provided as an a priori sample size calculation was not performed in a feasibility trial. Linear regression adjusting for baseline values of the outcomes were used to estimate the treatment effect with 95% CIs for the secondary outcomes.

## Results

**Figure 2:**
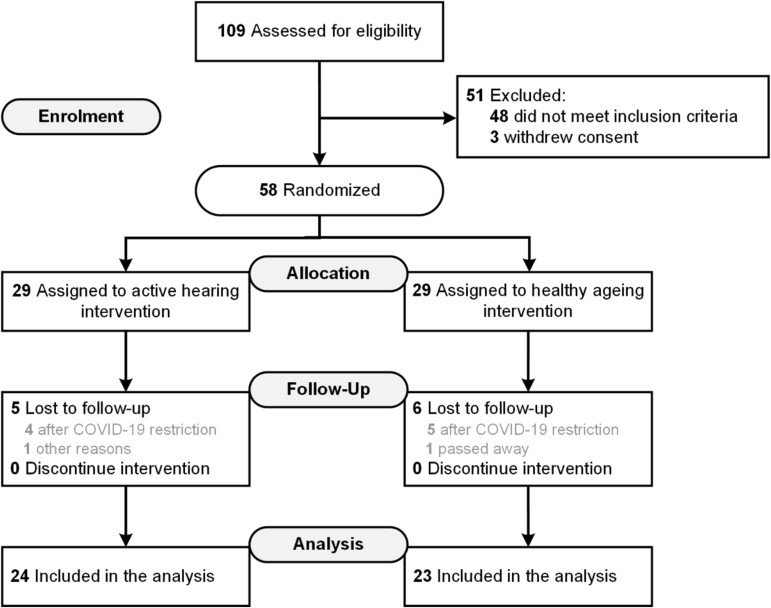
CONSORT Summary of recruitment and follow-up of participants in the study.

One hundred and nine participants were assessed and 58 were recruited and randomised from 27 October 2018 to 20 March 2020 (Fig. 2). From that date, trial recruitment paused and eventually stopped due to the first UK COVID-19 lockdown. Only two communication partners were recruited into the study. Baseline demographic and clinical data are shown in Table 1.

**Table 1:** Baseline demographic and clinical characteristics of 58 participants with MCI and hearing loss.

	Intervention	Control
	Mean (SD) /N (%)	Mean (SD) /N (%)
Demographics Age at screening (years)	79.9 (7.4)	77.8 (6.4)
Female gender	9 (32%)	11 (41%)
White British or other white background	22 (76%)	23 (79%)
Hearing		
Have you ever used hearing aids (single question)	11 (38%)	12 (41%)
Any self-perceived hearing loss (single question)	19 (66%)	18 (62%)
Any self-perceived hearing loss (single question)	19 (66%)	18 (62%)
At least moderate hearing handicap (HHIE-S ≥ 10)	18 (61%)	18 (61%)
PTA 0.5–4 kHz: right ear (dB HL)	37.4 (10.2)	37.1 (17.6)
PTA 0.5–4 kHz: left ear (dB HL)	39.9 (11.5)	39.9 (16.2)
QuickSIN average (dB SNR)	9.2 (3.8)	8.3 (6.5)
Medical history		
Any cardio-vascular condition	19 (68%)	23 (85%)
Diabetes	3 (11%)	10 (37%)
High blood pressure	13 (46%)	18 (67%)
Ischaemic heart disease	8 (29%)	2 (7%)
Stroke	5 (18%)	6 (22%)
Ever smoked	20 (71%)	19 (70%)
Alcohol intake (units/week)	4.1 (5.8)	8.5 (14.4)
Falls (times in the last 6 months)	0.6 (1.0)	1.2 (3.7)
Communication partner’s participation	2 (10%)	0 (0%)

HHIE-S, Hearing Handicap Inventory for the Elderly Screening Version; N, number of participants; PTA, pure tone average; QuickSIN, Quick Speech-In-Noise test. SD, standard deviation. SNR: Speech-to-noise ratio.

### Primary outcomes

All primary measures met the pre-specified target feasibility and acceptability benchmarks (see Table 2). Regarding the feasibility goals, of 109 consented participants, 61 met the eligibility criteria, and 58 of these [95%, (86%, 99%)] were recruited and randomised, 29 to each group. Forty-seven out of 58 participants [81% (69%, 90%)] were followed-up at 6 months and outcome measures were obtained from them.

**Table 2:** Hearing aid outcomes of TACT hearing intervention in MCI populations with hearing loss

Outcomes	Intervention group	Control group	% Difference [95% CI]
Number randomised	29	29	-
Intervention completion rate (2 visits or more)	24/29 (83%)	21/29 (72%)	+10.3% [−11%, 32%]
^a^Hearing aids outcomes at 6 m			
Fitted with hearing aids	24/29 (83%)	6/29 (21%)	+62% [42%, 82%]
			
Self-reported use (IOI-HA)			
Mean daily use in hours (SD)	5.3 (4.6)	1.8 (4.2)	-
Daily use >0 hr	18/24 (75%)	5/23 (22%)	+53% [29%, 77%]
*^a^*Daily use ≥4 hr	13/24 (54%)	3/23 (13%)	+41% [17%, 65%]

^a^Indicates the secondary outcomes; hr, hours; CI, confident interval; numbers are number of participants and percentage, except for daily hearing use in hours, with SD, standard deviation. IOI-HA: International Outcome Inventory for Hearing Aids [27].

Regarding the acceptability goals, 24 out of 29 participants (83%) completed two or more sessions of the active intervention, and 21 out 29 participants (72%) in the control group completed two or more sessions [difference + 3.4% (−17%, 23%)]. In the intervention group 18/24 (75%) self-reported daily hearing aid use at 6 months, whilst the proportion in the control group was 5/23 (22%), a difference of +53% (29%, 77%) favouring the active intervention.

### Secondary outcomes

We collected data face-to-face before lockdown, and by telephone after. See Table 2, intervention group average self-reported hearing aid use was 5.3 h overall (5.4 h telephone, 7/7 fitted; 5.2 h in-person, 16/17 fitted). Control group had 1.8 h overall (0.0 h telephone, 1/4 fitted; 2.2 h in-person, 5/19 fitted).

Most participants deemed the interventions to be somewhat or perfectly acceptable: 95% in the active intervention group, 89% in the control group, difference + 6% (−11%, 23%) (see Appendix 5 in the Supplementary Data section for the secondary outcomes with respect to acceptability). There were 6 serious adverse events, 3 in each group, and none deemed related to the intervention. One adverse event was recorded for a participant randomised to the intervention group. The participant reported experiencing itchiness whilst using the hearing aids; this was investigated by the trial team and the participant was referred for cerumen removal, which resolved the issue.

Secondary outcomes of cognition, self-reported hearing disability, depression, quality of life, loneliness, activities of daily living (functional independence, physical strength) were successfully gathered at baseline and at 6 months, allowing for pilot data on intervention effects (see Table 3).

**Table 3:** Baseline and 6-month measures of cognition, hearing disability, depression, quality of life, loneliness, social functioning and physical function outcomes in both groups and the intervention effect.

Outcomes	Baseline		6-month follow up		Intervention effect^a^ (95% CI)
	Intervention mean (SD)	Control mean (SD)	Intervention mean (SD)	Control mean (SD)	
Cognition					
ACE-III total score	78.3 (13.0)	78.6 (16.7)	80.9 (10.6)	80.8 (15.5)	+1.2 (−1.9, 4.2)
Memory	17.8 (6.0)	18.9 (6.3)	18.7 (5.8)	19.6 (6.6)	+0.5 (−1.4, 2.4)
TMT-A—processing speed	64.8 (52.4)	56.1 (41.6)	57.9 (42.0)	45.2 (20.3)	−2.7 (−14.3, 9.0)
TMT-B—cognitive flexibility	141.7 (63.8)	123.4 (56.2)	142.3 (60.1)	135.9 (64.0)	−8.6 (−33.9, 16.6)
DWRT—total words recalled	2.7 (2.3)	3.4 (1.8)	3.2 (2.9)	2.9 (2.2)	+1.0 (−0.3, 2.3)
Hearing disability					
HHIE-S	14.6 (12.4)	13.2 (9.8)	10.1 (11.1)	12.8 (9.0)	−3.3 (−9.7, 3.1)
Depression					
GDS	3.3 (2.6)	3.9 (3.5)	2.3 (1.9)	5.1 (4.3)	−2.1 (−4.0, −0.3)
Quality of life					
SF-36—general health	58.8 (21.9)	54.6 (25.0)	56.9 (22.7)	47.8 (26.4)	+4.8 (−5.3, 14.8)
EQ-5D—your health today	68.3 (23.7)	64.9 (20.9)	70.8 (21.2)	67.4 (20.4)	+0.7 (−10.6, 11.9)
Loneliness					
UCLA loneliness scale score	33.4 (10.0)	37.0 (9.7)	29.9 (5.5)	36.9 (10.0)	−1.8 (−7.8, 4.3)
Social functioning					
SF-DEM total score	27.6 (6.5)	29.1 (6.1)	27.3 (5.6)	29.4 (4.9)	−0.7 (−3.5, 2.0)
Functional independence					
IADL score	3.7 (0.6)	3.7 (0.7)	3.8 (0.4)	3.9 (0.3)	−0.1 (−0.3, 0.2)
Physical strength					
Grip strength average	21.7 (10.0)	22.1 (8.9)	23.5 (8.6)	22.7 (7.6)	0.8 (−1.4, 3.0)

^a^Intervention effects were calculated using linear regression models adjusted for baseline outcome value. ACE-III, Addenbrooke’s Cognitive Examination IIIDWRT, Delayed Word Recall Test; EQ-5D, EuroQol-5 Dimension; GDS, Geriatric Depression Scale; HHIE-S, Hearing Handicap Inventory for the Elderly Screening Version; IADL, Brody instrumental activities of daily living scale; N, number of participants; SF-36, 36-Item Short Form Survey; SFDEM, Social Functioning in Dementia Scale; TMT-A & B, Trail Making Test Parts A & B.

## Discussion

The findings in this pilot trial met pre-specified targets for feasibility of recruitment and assessment procedures, and for acceptability of the intervention. Thus, our findings pave the way for a fully-powered multicentre RCT focusing on longer-term cognitive outcomes in people with MCI and hearing loss. This trial makes several important contributions, including focusing on people with MCI, and demonstrating that it is feasible to significantly increase hearing aid usage in this population. Lastly, the similarity of our control group outcomes to real-world estimates from the UK enhances the validity of our findings and supports their potential application in clinical settings.

Our study was conducted within the UK NHS, which importantly provides free hearing care, whereas access to hearing aids and related services in the US may differ due to variations in insurance coverage and out-of-pocket costs. As a result, our study provides valuable insights specific to the UK healthcare system and offers external validation of the US ACHIEVE findings [7, 8] in a different healthcare context.

Self-reported hearing aid use at 6-month follow-up was 75% for any daily use in the hearing intervention group compared to 22% in the control group, where participants and GPs were informed of the hearing loss and encouraged their GPs to consider referral to standard audiological pathways. Data collection format did not appear to affect reported hearing aid usage in the intervention group. For the control group, the low number of participants using hearing aids, particularly amongst those interviewed by phone, makes it difficult to draw firm conclusions. However, available data suggests that the format likely had minimal impact on reported usage in this group as well. For reference, hearing aid adoption rates among those aged 55 and over with self-reported hearing loss ranged from 43% to 65% in 2022 in the UK. However, in this online panel data, it is likely that people with MCI were underrepresented [10].

Overall, our findings suggest that our TACT hearing intervention could be useful in supporting hearing aid fitting and use in people with cognitive difficulties who have specific challenges with accessing hearing healthcare and with learning how to regularly use hearing aids [28].

The TACT intervention exceeds typical audiological practice to ensure adherence in people with MCI, for whom standard pathways often fail. This intensive approach demonstrated successful adoption and sustained hearing aid use. Our findings suggest that the main active component of the intervention was increasing the rate of hearing aid fittings compared to standard pathways. Amongst those fitted, hearing aid use was similar between groups (see Appendix 6 in the Supplementary Data for the full details), though the number of controls who received hearing aids was small. Consequently, post-fitting support could potentially be reduced in a fully-powered RCT without compromising effectiveness.

Our study reflects the real-world challenges of accessing NHS hearing care, where GP referrals to audiology services may not always be straightforward, and self-referrals are an alternative. Whilst we wrote to GPs recommending referral and informed participants of self-referral options, we did not collect detailed data on the pathway, other than whether participants were fitted with hearing aids, and their self-reported usage. This limitation highlights the need for further research to understand and improve referral processes for people with MCI.

Our results suggest that substantial gains in hearing aid use could potentially be achieved by improving NHS pathways to ensure more people with MCI are fitted with hearing aids. This is particularly important as people with MCI may be at higher risk of not being fitted due to cognitive difficulties, such as remembering appointments.

Interestingly, participants’ awareness of being in the trial and their knowledge of the link between dementia and hearing loss may have improved adherence in the control group (see Appendix 6 in the Supplementary Data section for the full details of self-reported hearing aid use amongst those who were fitted with hearing aids between groups). Several participants reported that knowing about this potential link was a strong motivator for getting and using their hearing aids.

The trial was not powered to detect differences in cognitive or other secondary outcomes. However, the observed 1.2-point difference in ACE-III total scores between groups at 6 months suggests a potential cognitive effect of the hearing intervention, warranting investigation in a fully-powered RCT. Observational evidence suggests hearing aid use may lower dementia risk by 27% in people with MCI [29]. The ACHIEVE trial’s population-based higher risk subsample, similar to our participants in age and cognitive scores, showed reduced 3-year cognitive decline with hearing intervention [7, 8].

This trial had limitations. First, the first UK COVID-19 lockdown resulted in a halt in recruitment and did not permit delivery of the home-based visits after lockdown, leading to a reduced recruited number (58 out of intended 76), and contributing to reduced adherence to the active and control intervention, and to loss to follow-up. Future fully-powered RCT will require to incorporate modifications to simplify recruitment and support long-term adherence, including prioritising hearing aid fitting given the results of our trial. Second, whilst the hearing intervention used in this trial was effective in improving adherence, it is resource intensive. There are 4 sessions conducted over 3 months, of which 2 sessions are delivered by a qualified audiologist. A future trial will need to address the cost-effectiveness of the intervention. Third, our trial participants may represent a group that is more motivated to use hearing aids than those who declined participation. This motivation could have influenced our estimates of hearing aid use in both the intervention and control groups. However, we believe this is unlikely to have affected the observed difference between groups, as the effect of the intervention is based on relative comparisons rather than absolute levels of hearing aid uptake. Fourth, we employed an intention-to-treat approach for participants who did not adhere to the treatment but remained in the trial, using available outcome data. No imputation was performed for participants lost to follow-up, as it would have been challenging to produce reliable imputations given the small sample size and the number of participants not fitted with or using hearing aids. Furthermore, it was also not practicable to blind participants or those delivering the intervention, and whilst outcome assessment was done by assessors not involved in the intervention of that participant and blinded to allocation, assessors could surmise allocation if participants wore hearing aids. We employed self-reported hearing aid use due to practical difficulties in obtaining consistent data logging from the heterogeneous hearing aids fitted in the control group. Additionally, we were only able to recruit two communication partners to the trial, as many participants did not rely on support or had little regular contact with others. Another limitation is the chance imbalances between the baseline group characteristics. The intervention group was 2-year older on average with a higher proportion of ischaemic heart disease, whilst the control group had higher proportion of diabetes and high blood pressure. We think it is unlikely that such imbalances have impacted the findings of this pilot trial about differences in hearing aid use between arms. However, given that a fully-powered RCT would likely have a primary cognitive outcome, we suggest that sensitivity analyses adjusting for these baseline variables should be considered as part of the analytic plan for future trials. Lastly, our intervention lasted 3 months on average, with good adherence at 6 months follow-up. However, a fully-powered RCT to measure cognitive decline differences between groups requires at least 1 year. Evidence suggests behavioural changes sustained for 6 months often persist. Our planned fully-powered RCT may include post-intervention check-ins and booster sessions to reinforce hearing aid adherence [30].

## Conclusions

Randomisation to a personalised hearing support with hearing aid provision versus control was feasible and acceptable in older adults with MCI, leading to increased hearing aid fitting and use. Study findings also demonstrated a positive signal of hearing intervention on measures of cognition, mood and quality of life at 6 months, paving the way for a fully-powered multicentre RCT focusing on cognitive outcomes.

## Supplementary Material

aa-24-0839-File002_afaf004R

## Data Availability

Where ethically feasible, we will inform readers about data accessibility, including links to relevant datasets when available.
