# Supplementary material for: Early detection and management of hearing loss to reduce dementia risk in older adults with mild cognitive impairment: findings from the treating auditory impairment and cognition trial (TACT)
Source: Age Ageing. 2025 Jan 21;54(1):afaf004. doi: 10.1093/ageing/afaf004 (PMC11747994; doi:10.1093/ageing/afaf004)
Supplement: aa-24-0839-File002_afaf004R [file aa-24-0839-file002_afaf004r.docx]

**Early detection and management of hearing loss to reduce dementia risk in older adults with mild cognitive impairment: Findings from the Treating Auditory impairment and Cognition Trial (TACT)**

**Contents list**

# Appendix 1. The assessments used in the study

# Appendix 2. The TIDieR (Template for Intervention Description and Replication) Checklist

# Appendix 3. The four-session intervention in two groups

# Appendix 4. Pre-specified trial targets for recruitment, randomisation, retention, intervention acceptability, and hearing aid use differences between groups, with their expected 95% confidence interval based on projected sample size.

# Appendix 5. Secondary outcomes with respect to acceptability

# Appendix 6. Self-reported hearing aid use among those who were fitted with hearing aids between groups

# Appendix 1. The assessments used in the study.

| **Test name** | **Brief instruction** |
| --- | --- |
| *1. Audiometric assessments* | |
| **Ambient Noise Check** *(S)* | Before performing audiological procedures, the free-field testing room ambient noise was measured in dBA using a sound level meter. If this value was greater than 50 dBA, the testing environment was considered too noisy and remedial measures were taken to reduce it. |
| **+Client Orientated Scale of Improvement, COSI** *(TACT intervention group only; A, B, C, D, FU, ED*)* | The COSI, developed by National Acoustic Laboratories[1] is used to identify up to five specific situations that each participant would like to have improved by the hearing intervention. These situations can be listening situations, or they can be emotional or social situations. The identified situations are categorized into one of 16 standard categories. |
| **-HearCheck Screening Audiometry** *(S)* | Hearing screening was conducted using a calibrated handheld device (HearCheck; Siemens)[2]. Each participant was presented with a series of 3 higher frequency sounds (3 kHz) at decreasing intensities (75, 55, and 35 dB) and 3 midfrequency sounds (1 kHz) at decreasing intensities (55, 35, and 20 dB) administered to each ear separately. The participant indicated if they heard a sound by raising their hand. |
| **-Hearing Handicap Inventory for the Elderly, HHIE-S** *(self-report hearing disability; BL, FU, ED*)* | The HHIE-S (screening version) was developed by the American Speech-Language-Hearing Association (ASHA)[3], which measures perceived hearing handicap. Each question was scored as yes (4 points), sometimes (2 points), or no (0 points). The possible scores ranged from 0 (no handicap) to 40 (maximum handicap). The higher the scores, the greater was the handicapping effect of the hearing loss. |
| **-Quick Speech in Noise, QuickSI** *(S)* | QuickSIN was used to assess the ability to perceive speech in noise[4]. There are 12 lists of six sentences, each possessing five keywords that must be repeated by the subject to receive a score. The signal-to-noise (SNR) ratio starts at +25 in the first sentence and then decreases to 0 in 5-dB steps over the course of the six sentences. The final score is calculated as the SNR Loss score (in dB) using the correctly repeated keywords from the presented sentences with the following equation: SNR Loss score = 25.5 – (# of words correct). The SNR Loss score indicates the dB increases in SNR required by a hearing-impaired person to understand speech in noise, compared to normal hearing listeners. Therefore, the smaller the SNR Loss, the better the participant performed. The test was performed using an Interacoustics Callisto AC440. Each participant was presented with one practice list and two lists of sentences binaurally using DD-45 headphones at 70dBHL sound level. The final SNR Loss score was the average score of the two lists. |
| **Otoscopy** *(S, B*, D*)* | Otoscopy was performed prior to administering audiological procedures by a trained audiologist, following the British Society of Audiology (BSA) Recommended Procedure for Ear Examination (2016) [5]. |
| **Otological History** *(S)* | An otological history was taken for each participant to ascertain if they met the criteria for onward medical referral according to British Academy of Audiology criteria and therefore would be unsuitable to take part in the study. |
| **+Phoneme Recognition in Quiet testing, PRQ** *(S)* | PRQ testing was performed by a trained audiologist following the Manual of Operating Procedures using an Interacoustics Callisto AC440. Each participant was administered an AB word list consisting of 10 words using DD-45 headphone (plus audiocups). The initial presentation level was calculated using the PTA 2 kHz threshold (2 kHz threshold < 50 dB HL: 25 dB SL, 2 kHz threshold 50-55 dB HL: 20 dB SL, 2 kHz threshold 60-65 dB HL: 15 dB SL, 2 kHz threshold 70-75 dB HL: 10 dB SL). If they scored 60% phonemes correct or greater the test was stopped, if they scored less than 60% the presentation level was increased by 10dB, and a different 10-word list presented until they reached a score 60% (if they could not reach 60% they were considered illegible for the study). |
| **- Pure-Tone Audiometry, PTA** *(S)* | Participants underwent PTA using an Interacoustics Callisto AC440 audiometer performed by an experienced audiologist at the participant’s home, using gold-standard procedure [6]. Air-conduction thresholds were measured at frequencies 250, 500, 1000, 2000, 3000, 4000, 6000, 8000 Hz using DD-45 headphones (plus noise reduction audiocups) for both ears. Bone-conduction thresholds were also measured at 500, 1000, 2000, 4000 Hz using B-71 bone transducer. Hearing loss was defined for this trial (four-frequency pure tone average (0.5, 1, 2, 4 kHz) in the better-hearing ear of ≥ 25 dB HL and <70 dB HL or a pure tone audiometric threshold at 4 KHz in the better ear of ≥ 30 decibels dB HL). |
| **-Shoebox Screening Audiometry** *(S)* | Shoebox screening audiometry was conducted at the participants home using a calibrated table-based device (Shoebox) at frequencies 500, 1000, 2000, 4000 Hz using DD-450 headphones [7]. The participant was instructed to complete the automatic test by listening to ascending-descending pure tones and indicating if they heard the tone. If the participant struggled to complete the task it was switched to an assisted mode. |
| **Tuning Fork Test** *(S*)* | If the participant was unable to complete tympanometry, for example if an adequate probe seal could not be obtained, a Rinne Tuning Fork test was performed [8] to rule out a conductive hearing loss. A 512 Hz tuning fork was struck and alternatively placed on the mastoid and close to the entrance to the ear canal. The participant was asked which placement produced the louder sound, with a positive (bone conduction quieter than air) or negative (bone conduction louder than air) Rinne result (suggesting conductive hearing loss). This was repeated for both ears. |
| **Tympanometry** *(S)* | Tympanometry was performed on all participants with appropriate otoscopic findings to test the condition of the middle ear as a function of ear canal pressure using an Interacoustics Titan tympanometer. It was completed by a trained audiologist. Follow BSA Recommended Procedure for Tympanometry (2013) [9]. Daily calibration of the tympanometer with a 2-cc cavity was performed to ensure that the equipment was performing up to the manufacturer-specified standards.  The dementiaentional 226 Hz pure tone with pump speed setdementia medium (200 daPa/s) and pressure range from positive +300 daPa to negative −300 daPa were selected. The recordings were taken once in each ear for every participant. |
| *2. Neurocognitive assessments* | |
| **-Speech audibility**  **Procedure**  *(BL, FU, ED*)* | A standardised procedure for speech audibility evaluation was employed to prevent bias by assessing the participants' ability to comprehend speech in a quiet setting prior to the formal assessment. |
| **+Addenbrooke Cognitive Examination-III, ACE-III** *(BL, FU, ED*)* | The ACE-III is a cognitive test assessing five cognitive domains: attention, memory, verbal fluency, language, and visuospatial abilities [10]. The total score is 100, with higher scores indicating better cognitive functioning. A score of 82/100 is accepted as the cut-off for an indication of possible dementia. |
| **-Trail Making Test, TMT** *(BL, FU, ED*)* | The TMT assesses visual scanning, graphomotor speed and executive function; results are reported for part A and B as the time taken to complete the tasks (results reported in seconds) [11]. |
| **+Delayed Word Recall Test, DWRT** *(BL, FU, ED*)* | The DWRT consists of the participant repeating and encoding 10 presented words, a filled delay of 5 minutes, and then the delayed free recall section [12]. |
| *3. Depression* | |
| **-Geriatric Depression Scale, GDS** *(BL, FU, ED*)* | Mood was assessed using the GDS [13]; a 15-item ‘yes/no’ questionnaire which measures self-reported depression in older adults. A score of 5 or higher indicates the presence of depressive symptoms. |
| *4. Quality of Life* | |
| **+36-Item Short Form Survey, SF-36** *(BL, FU, ED*)* | The SF-36 [14] consists of 36 questions, with 8 subscales, measuring health-related quality of life. These domains include physical functioning, mental health, general health, social functioning, bodily pain, vitality, role-physical and role-emotional. Higher scores on all subscales are indicative of better functioning and overall health. |
| **+E–roQol - 5 Dimension, EQ-5D-3L** *(BL, FU, ED*)* | The EQ-5D-3L [15] assesses 5 aspects of health: mobility, self-care, usual activities, pain/discomfort, and anxiety/depression. Each dimension includes 3 levels which indicate different levels of severity (no problems, some problems, extreme  problems). The EQ-VAS is a 0-100 scale where the participant was asked to rate their subjective health state that day, with higher numbers indicating better self-reported health. |
| *5. Loneliness* | |
| **-UCLA Loneliness Scale** *(BL, FU, ED*)* | Loneliness was measured using the UCLA Loneliness Scale (Version 3) [16], which consists of 20-items measuring subjective feelings of social isolation and loneliness. The total score is 80, with higher scores indicating higher levels of self-perceived loneliness. |
| *6.. Social functioning* | |
| **+Social Functioning in Dementia Scale, SF-DEM** *(BL, FU, ED*)* | This scale includes 2 sections [17]; the first consists of 11 questions of self-reported social activities and the second consists of 6 questions of self-reported personal relationships. The total score of the combined sections is 51, with higher scores suggestive of higher social functioning. |
| *7. Functional independence* | |
| **-Brody instrumental activities of daily living scale, IADL** *(BL, FU, ED*)* | This consists of 8 domains of function featuring complex tasks [18]. Only 4 sections were used (ability to use telephone, mode of transportation, responsibility for own medications, and ability to handle finances) as these have been shown to be most predictive of future progression to dementia, accumulating a possible total score of 4. Higher scores indicate higher levels of functioning. |
| *8. Physical strength* | |
| **+Grip dynamometer** *(BL, FU, ED*)* | A grip dynamometer was used to measure physical strength [19] . |

A '+' symbol denotes that higher values (volumes, scores, numbers) are favourable, while a '-' symbol signifies that lower values are preferable; * Indicates that the conduction of the test is optional; A, B, C, D refer to intervention session A (week 3 – 4), session B (week 5 – 6), session C (week 7 – 8), session D (week 9 – 10); BL, baseline visit (day 0-14); FU, follow-up visit (at 6 months); ED, early discontinuation. S, screening visit (day 0); The communication partner of the patient (if applicable) was evaluated using the SF-36, EQ-5D, SF-DEM, and IADL at baseline and 6-month follow-up visits.

# **
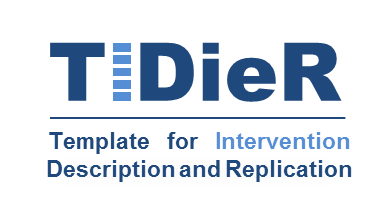
**Appendix 2. The TIDieR (Template for Intervention Description and Replication) Checklist

**The TIDieR (Template for Intervention Description and Replication) Checklist*** [20]**:**

Information to include when describing an intervention and the location of the information

| **Item number** | **Item** | **Where located **** | |
| --- | --- | --- | --- |
|  |  | Primary paper  (page or appendix  number) | Other ^†^ (details) |
|  | **BRIEF NAME** |  |  |
| **1.** | Provide the name or a phrase that describes the intervention. | Page 6 | Protocol: 3. Background and Rationale |
|  | **WHY** |  |  |
| **2.** | Describe any rationale, theory, or goal of the elements essential to the intervention. | Page 6-10, Appendix 3 | Protocol: 3. Background and Rationale |
|  | **WHAT** |  |  |
| **3.** | Materials: Describe any physical or informational materials used in the intervention, including those provided to participants or used in intervention delivery or in training of intervention providers. Provide information on where the materials can be accessed (e.g. online appendix, URL). | Page 6-10,  Appendix 3 | Protocol: 5. Trial design |
| **4.** | Procedures: Describe each of the procedures, activities, and/or processes used in the intervention, including any enabling or support activities. | Page 6-8,  Appendix 3,  Figure 1 | Protocol: 7. Product/  Interventions |
|  | **WHO PROVIDED** |  |  |
| **5.** | For each category of intervention provider (e.g. psychologist, nursing assistant), describe their expertise, background and any specific training given. | Page 6-10, Figure 1 | Protocol: 8. Trial procedures |
|  | **HOW** |  |  |
| **6.** | Describe the modes of delivery (e.g. face-to-face or by some other mechanism, such as internet or telephone) of the intervention and whether it was provided individually or in a group. | Page 6-10, Figure 1 | Protocol: 8. Trial procedures |
|  | **WHERE** |  |  |
| **7.** | Describe the type(s) of location(s) where the intervention occurred, including any necessary infrastructure or relevant features. | Page 6-10, Figure 1 | Protocol: 8. Trial procedures |
|  | **WHEN and HOW MUCH** |  |  |
| **8.** | Describe the number of times the intervention was delivered and over what period of time including the number of sessions, their schedule, and their duration, intensity or dose. | Page 6-10, Appendix 3,  Figure 1 | Protocol: 8. Trial procedures |
|  | **TAILORING** |  |  |
| **9.** | If the intervention was planned to be personalised, titrated or adapted, then describe what, why, when, and how. | Page 8-10, Figure 1 | Protocol: 7. Product/  Interventions |
|  | **MODIFICATIONS** |  |  |
| **10.^ǂ^** | If the intervention was modified during the course of the study, describe the changes (what, why, when, and how). | Page 9, Figure 1 | Protocol: *Figure 4. (COVID-19)* |
|  | **HOW WELL** |  |  |
| **11.** | Planned: If intervention adherence or fidelity was assessed, describe how and by whom, and if any strategies were used to maintain or improve fidelity, describe them. | Page 12 | Protocol: 11.6 Statistical analysis |
| **12.^ǂ^** | Actual: If intervention adherence or fidelity was assessed, describe the extent to which the intervention was delivered as planned. | Page 12-15 | N/A |

** **Authors** - use N/A if an item is not applicable for the intervention being described. **Reviewers** – use ‘?’ if information about the element is not reported/not sufficiently reported.

† If the information is not provided in the primary paper, give details of where this information is available. This may include locations such as a published protocol or other published papers (provide citation details) or a website (provide the URL).

ǂ If completing the TIDieR checklist for a protocol, these items are not relevant to the protocol and cannot be described until the study is complete.

* We strongly recommend using this checklist in conjunction with the TIDieR guide (see *BMJ* 2014;348:g1687) which contains an explanation and elaboration for each item.

* The focus of TIDieR is on reporting details of the intervention elements (and where relevant, comparison elements) of a study. Other elements and methodological features of studies are covered by other reporting statements and checklists and have not been duplicated as part of the TIDieR checklist. When a **randomised trial** is being reported, the TIDieR checklist should be used in conjunction with the CONSORT statement (see [www.consort-statement.org](http://www.consort-statement.org)) as an extension of **Item 5 of the CONSORT 2010 Statement.** When a **clinical trial** **protocol** is being reported, the TIDieR checklist should be used in conjunction with the SPIRIT statement as an extension of **Item 11 of the SPIRIT 2013 Statement** (see [www.spirit-statement.org](http://www.spirit-statement.org)). For alternate study designs, TIDieR can be used in conjunction with the appropriate checklist for that study design (see [www.equator-network.org](http://www.equator-network.org)).

# Appendix 3. The four-session intervention in two groups

| **Session** | **Topic**  (TACT intervention group vs Control group) | **Content** |
| --- | --- | --- |
| A  *(W3–4)* | **TACT group:** Orientation and goal setting (RA or Research Audiologist)  **Control group:** Lower systolic blood pressure (RA) | - Both groups received an orientation and overview of their respective interventions. - The intervention group focuses on daily routines, communication needs, hearing aid usage reminders, and watching a video on what to expect with hearing aids. - While the control group discussed lowering blood pressure. - Both groups filled out a questionnaire on hearing aid help needs and an adverse events questionnaire. |
| B  *(W5–6)* | **TACT group:** Hearing aid fitting and training (Research Audiologist)  **Control group:** Eat a healthy diet (RA) | - The intervention group reviewed hearing aid goals and discussed hearing aid expectations. Hearing aids were fitted. The control group discussed healthy diet and data on self-reported hearing aid use was collected, if applicable. - Both groups again filled out an adverse events questionnaire. |
| C  *(W7–8)* | **TACT group:** Review of hearing aid use and communication strategies (RA)  **Control group:** Be physically active (RA) | - The intervention group reviewed hearing aid usage and integration into their daily routine, as well as communication strategies. The control group discussed being physically active. - Both groups self-reported on hearing aid use and need for help (only when applicable in the control group) and completed an adverse events questionnaire. |
| D  (*W9–10)* | **TACT group:** Hearing aid optimisation and progress review (RA or Research Audiologist)  **Control group:** Maintain healthy bones, joints and muscles (RA) | - The intervention group evaluated hearing aid use, reviewed routine integration, and fine-tuned hearing aids, if required. Participants also rated the hearing intervention and gave feedback. - The control group discussed maintaining healthy bones, joints, and muscles. Participants rated the healthy ageing intervention and gave feedback. - Both groups self-reported on hearing aid use and need for help (only when applicable in the control group) and completed an adverse events questionnaire. |

RA, research assistant; W, week. C2Hear videos [21] was used in TACT group during Sessions A-D, COSI [1] goals set at session A were reviewed at sessions B through D, as well as during follow-up assessments.

# Appendix 4. Pre-specified trial targets for recruitment, randomisation, retention, intervention acceptability, and hearing aid use differences between groups, with their expected 95% confidence interval based on projected sample size.

| The goals set in this pilot trial | The 95% CI of the expected estimates  (with n=76) |
| --- | --- |
| (i) Feasibility |  |
| 1. Recruitment and randomisation:   50% of eligible participants will be randomised | 39% - 61% |
| 1. Retention (with complete follow-up at 6-month):   80% randomised participants | 71% - 89% |
| (ii) Acceptability |  |
| 1. Acceptability of hearing intervention:   80% of intervention group completing ≥2 intervention sessions | 71% - 89% |
| 1. Daily hearing aid adherence (> 0 hour):   50% difference between groups at the end of trial | 31% - 69% |

CI, confidence interval; n, numbers of participants. The sample size was calculated based on the Sample Sizes for Clinical, Laboratory and Epidemiology Studies [22]*.*We pre-defined the following criteria for proceeding to a fully powered RCT: (A) Proceed to a fully powered RCT: Recruited >50% (95% CI: 39% - 61%) of eligible participants; retained >80% (95% CI: 71% - 89%) of participants at 6 months; completed >2 hearing aid support sessions for >80% (95% CI: 71% - 89%) at 6 months; achieved >50% (95% CI: 31% - 69%) increase in daily hearing aid usage, favouring the intervention group. (B) Proceed to a fully powered RCT with amendments: Any estimates that fall up to 10% below the lower limit of the target CI. (C) Do not proceed to a fully powered RCT: Any estimates that fall below the threshold described under (B).

# Appendix 5. Secondary outcomes with respect to acceptability

| **Secondary outcome measures** | **Results** |
| --- | --- |
| **Acceptability** |  |
| 1. *Participants refused or*   *discontinued* | There were 11 participants lost to follow-up (n = 6 in the intervention and n = 5 control groups), with none related to dissatisfaction with the intervention. |
| 1. *Participants fitted with*   *hearing aids in the*  *intervention group* | There were 24 participants (83%) fitted with hearing aids in the intervention group. |
| 1. *Adverse or serious adverse*   *events* | There were 6 serious adverse events (n = 3 in the intervention group and n = 3 in the control group), with none deemed to be related to the intervention, and 1 adverse event within the active intervention group |
| *(4) Satisfaction survey* |  |
| Somewhat or perfectly  acceptable | 95% and 89% participants in the intervention and the control groups (difference: 6% [95% CI= -11%, 23%]). |
| Neutral or somewhat or  totally unacceptable | 5% and 11% participants in the intervention and the control groups (difference: 6% [95% CI= -23%, 11%]). |

CI, confidence interval

# Appendix 6. Self-reported hearing aid use among those who were fitted with hearing aids between groups

| **Outcomes** | **Intervention**  **group** | **Control**  **group** | **% Difference**  **[95% CI]** |
| --- | --- | --- | --- |
|  |  |  |  |
| *Hearing aids outcomes at 6m* |  |  |  |
| Fitted with hearing aids | 24/29 (83%) | 6/29 (21%) | +62% [42%, 82%] |
|  |  |  |  |
| *Comparison of self-reported use (IOI-HA) between hearing aids users in two groups* |  |  |  |
| Daily use > 0 hour | 18/24 (75%) | 5/6 (83%) | -0.8% [-43%, 26%] |
| Daily use ≥ 4 hours | 13/24 (54%) | 3/6 (50%) | +4% [-41%, 49%] |

CI, confident interval. IOI-HA [23]: International Outcome Inventory for Hearing Aids.

**Reference**

1. Dillon H, Birtles G, Lovegrove R. Measuring the outcomes of a national rehabilitation program: Normative data for the Client Oriented Scale of Improvement (COSI) and the Hearing Aid User’s Questionnaire (HAUQ). *J Am Acad Audiol.* 1999; **10**: 67–79. <https://doi.org/10.1055/s-0042-1748459>
2. Parving A, Sørup Sørensen M, Christensen B, Davis A. Evaluation of a hearing screener. *Audiol Med.* 2008; **6**: 115–119. <http://doi.org/10.1080/16513860801995633>
3. Newman CW, Weinstein BE, Jacobson GP, Hug GA. The Hearing Handicap Inventory for Adults: Psychometric adequacy and audiometric correlates. *Ear Hear.* 1990; **11**: 430–433. <http://doi.org/10.1097/00003446-199012000-00004>
4. Killion MC, Niquette PA, Gudmundsen GI, Revit LJ, Banerjee S. Development of a quick speech-in-noise test for measuring signal-to-noise ratio loss in normal-hearing and hearing-impaired listeners. *J Acoust Soc Am.* 2004; **116**: 2395–2405. http://doi.org/[10.1121/1.1784440](https://doi.org/10.1121/1.1784440)
5. British Society of Audiology. Recommended procedure ear examination. 2016. West Lothian, UK: British Society of Audiology. Available at: [www.thebsa.org.uk](http://www.thebsa.org.uk) (12 April 2024, date last accessed).
6. British Society of Audiology. Recommended procedure pure-tone air-conduction and bone-conduction threshold audiometry with and without masking. 2018. West Lothian, UK: British Society of Audiology. Available at: [www.thebsa.org.uk](http://www.thebsa.org.uk) (12 April 2024, date last accessed).
7. Thompson GP, Sladen DP, Borst BJH, Still OL. Accuracy of a tablet audiometer for measuring behavioral hearing thresholds in a clinical population. *Otolaryngology–Head and Neck Surgery.* 2015; **153**: 838–842. <https://doi.org/10.1177/0194599815593737>
8. British Society of Audiology. Recommended procedure - Rinne and Weber tuning fork tests. 2016. West Lothian, UK: British Society of Audiology. Available at: [www.thebsa.org](http://www.thebsa.org) (12 April 2024, date last accessed).
9. British Society of Audiology. Recommended procedure: Tympanometry. 2013. West Lothian, UK: British Society of Audiology. Available at:[www.thebsa.org.uk](http://www.thebsa.org.uk) (12 April 2024, date last accessed).
10. Hsieh S, Schubert S, Hoon C, Mioshi E, Hodges JR. Validation of the Addenbrooke’s Cognitive Examination III in Frontotemporal Dementia and Alzheimer’s Disease. *Dement Geriatr Cogn Disord.* 2013; **36**: 242–250. <https://doi.org/10.1159/000351671>
11. Reitan’ RM. Validity of the Trail Making Test as an indicator of organic brain damage. *Percept Mot Skills.* 1958; **8**: 271–276. <https://doi.org/10.2466/pms.1958.8.3.271>
12. Knopman DS, Ryberg S. A verbal memory test with high predictive accuracy for dementia of the Alzheimer type. *Arch Neurol.* 1989; **46**: 141–145. <https://doi.org/10.1001/archneur.1989.00520380041011>
13. Sheikh JI, Yesavage JA. Geriatric Depression Scale (GDS): Recent evidence and development of a shorter version. *Clinical Gerontologist: The Journal of Aging and Mental Health.* 1986; **5**, 165–173. <https://doi.org/10.1300/J018v05n01_09>
14. Brazier JE, Harper R, Jones NMB *et al.* Validating the SF-36 health survey questionnaire: New outcome measure for primary care. *British Medical Journal.* 1992; **305**: 160-164. <https://www.jstor.org/stable/29716334>
15. Kind P, Hardman G, Macran S. UK population norms for EQ-5D. Working Papers, Centre for Health Economics, University of York. 1999. 172chedp. <https://ideas.repec.org/p/chy/respap/172chedp.html> (9 November 2023, date last accessed).
16. Russell DW. UCLA Loneliness Scale (Version 3): Reliability, validity, and factor structure. *J Pers Assess*. 1996; **66**: 20–40. <https://doi.org/10.1207/s15327752jpa6601_2>
17. Sommerlad A, Singleton D, Jones R, Banerjee S, Livingston G. Development of an instrument to assess social functioning in dementia: The Social Functioning in Dementia scale (SF-DEM). *Alzheimers Dement (Amst).* 2017; **7**: 88–98. <https://doi.org/10.1016/j.dadm.2017.02.001>
18. Pérès K, Chrysostome V, Fabrigoule C, Orgogozo JM, Dartigues JF, Barberger-Gateau P. Restriction in complex activities of daily living in MCI: Impact on outcome. *Neurology.* 2006; **67**: 461–466. <https://doi.org/10.1212/01.wnl.0000265318.46474.bf>
19. Roberts HC, Denison HJ, Martin HJ *et al.* A review of the measurement of grip strength in clinical and epidemiological studies: Towards a standardised approach. *Age Ageing.* 2011; **40**: 423–429. <https://doi.org/10.1093/ageing/afr051>
20. Hoffmann TC, Glasziou PP, Boutron I *et al.* Better reporting of interventions: template for intervention description and replication (TIDieR) checklist and guide. *BMJ*. 2014; **348**: g1687. <https://doi.org/10.1136/bmj.g1687>
21. Ferguson M, Brandreth M, Brassington W, Leighton P, Wharrad H. A randomized controlled trial to evaluate the benefits of a multimedia educational program for first-time hearing aid users. *Ear Hear.* 2016; **37**: 123-236. <http://doi.org/10.1097/AUD.0000000000000237>
22. Machin D, Campbell MJ, Tan SB, Tan SH. Sample sizes for clinical, laboratory and epidemiology studies, fourth edition. *John Wiley & Sons,* 2018. <http://doi.org/10.1002/9781118874905>
23. Cox RM, Alexander GC. The International Outcome Inventory for Hearing Aids (IOI-HA): Psychometric properties of the English version: El Inventario International de Resultados para Auxiliares Auditivos (IOI-HA): propiedades psicometricas de la version en ingles. *Int J Audiol.* 2002; **41**: 30–35. <http://doi.org/10.3109/14992020209101309>
